# Metastatic Merkel cell carcinoma (MCC) of pancreas and breast: a unique case

**DOI:** 10.1186/1477-7819-11-261

**Published:** 2013-10-07

**Authors:** Spiridon Vernadakis, Demetrios Moris, Agnes Bankfalvi, Nikolaos Makris, Georgios C Sotiropoulos

**Affiliations:** 1 Department of General, Visceral and Transplantation Surgery, University Hospital Essen, Hufelandstr. 55, Esse 45122, Germany; 2 Institute of Pathology and Neuropathology, University Hospital Essen, Hufelandstr. 55, Essen 45122, Germany; 3 1st Department of Surgery, Laikon General Hospital, National and Kapodistrian University of Athens, Athens 11527 Greece

**Keywords:** Merkel cell carcinoma, Merkel cell polyomavirus, Metastasis, Pancreatic tumors

## Abstract

Merkel cell carcinoma (MCC) is a rare potentially fatal skin tumor affecting older and immunosuppressed individuals. It is highly malignant with high rates of metastasis and poor survival.

We present a case of a 67-year-old woman with a palpable mass in the upper abdomen. An abdominal CT revealed a mass in the tail of the pancreas. Two weeks before, lumpectomy of a 3.5 cm tumor of the left breast had been performed. Histology showed a primary neuroendocrine carcinoma of the mammary gland. The patient’s medical history was significant for a 0.7 × 0.9 cm MCC removed from her left forearm 2.5 years ago. There was no evidence of vascular involvement or peritoneal disease and by all criteria was resectable. A somatostatin receptor scintigraphy showed an enhanced uptake in the pancreatic tail region. The tumor was immunohistochemically strong staining for synaptophysin and CD56. The diagnosis of a metastatic-MCC in the tail of the pancreas was made. Further histological investigation of the prior removed neuroendocrine breast tumor and the MCC of the left forearm confirmed neuroendocrine origin and identical histology to the previously resected MCC of the left forearm. In this article, we aim to highlight that MCC has the potential to spread even in unusual organs, such as pancreas or breast, and therefore a diligent follow-up should be applied in patients with MCC.

## Background

Merkel cell carcinoma (MCC) is a rare, potentially fatal skin tumor affecting older, mainly white people and younger immunosuppressed individuals [1]. It is a highly malignant tumor of the skin with high rates of metastasis and poor survival. Its incidence rate is rising and is currently approximately 0.6/100,000 cases per year [1,2]. We present a unique case of concomitant metastatic MCC to pancreatic tail and breast.

## Case presentation

**Figure 1:**
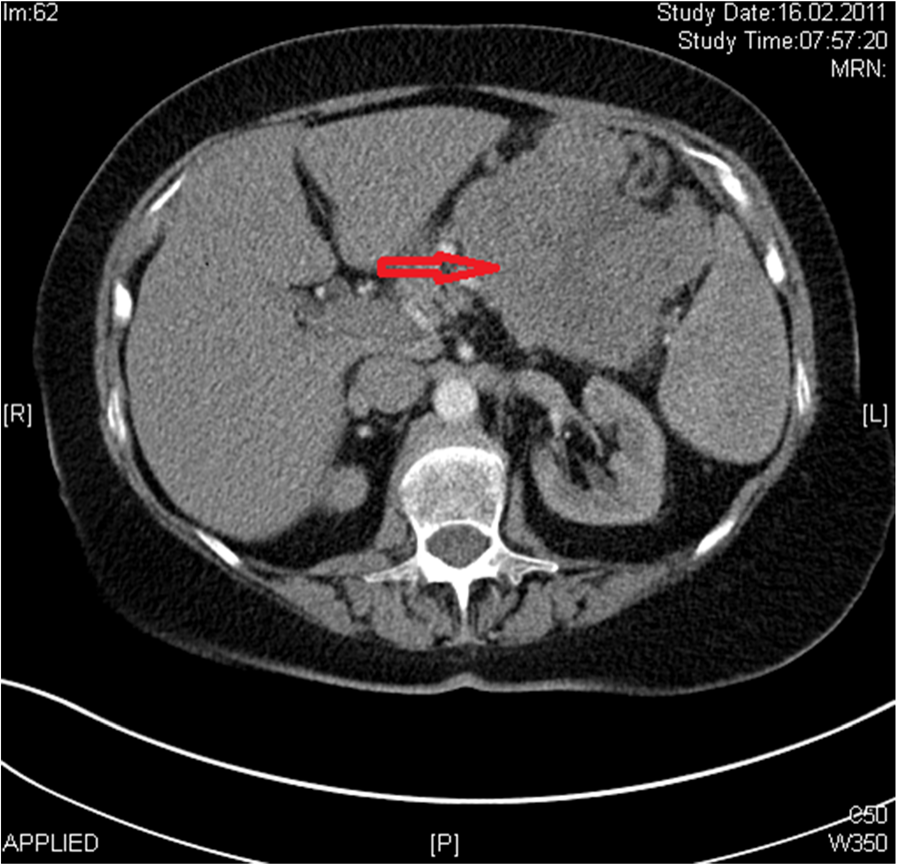
CT scan of the abdomen demonstrating a large mass in relation to the pancreatic tail.

**Figure 2:**
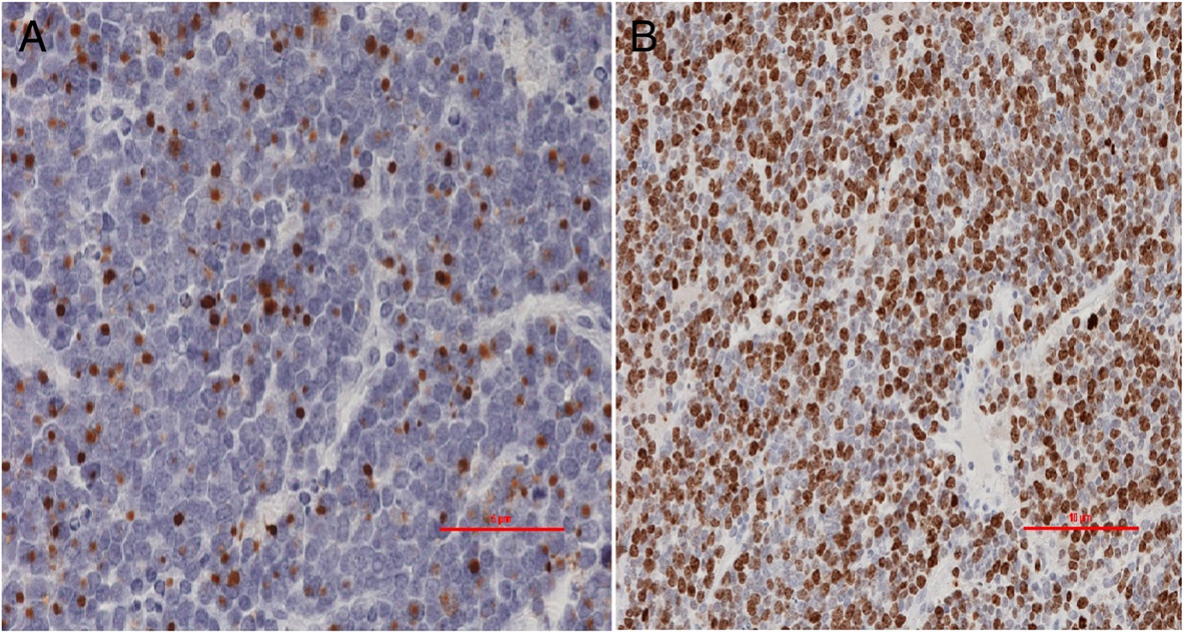
**Histology of the pancreatic tumor – MCC metastasis. (A)** Strong immunohistochemical staining against CK20 (original magnification × 400). **(B)** Strong proliferative activity Ki-67 of 80% (original magnification × 200).

A 67-year-old woman was referred to Department of General, Visceral and Transplantation Surgery of University Hospital Essen with a palpable mass in the upper abdomen. An abdominal CT revealed a large mass in the tail of the pancreas (10 × 14 cm) (Figure 1) without evidence of other peritoneal disease during staging investigation for a suspected carcinoma of the left breast. Two weeks previously, lumpectomy of a 3.5 cm tumor of the left breast had been performed by gynecologists. Sentinel lymph node examination was negative. Histology showed a primary poorly differentiated neuroendocrine carcinoma of the mammary gland. The patient’s medical history was significant for mediastinal sarcoidosis and a 0.7 × 0.9 cm MCC removed from her left forearm 2.5 years previous, which was Merkel cell polyomavirus (MCV) DNA positive after quantitative PCR. No viral DNA was found in peripheral blood. Postoperative adjuvant external beam radiation therapy of 52 Gy was given on the left forearm. Clinically, the patient reported abdominal discomfort and weight loss of 8 kg over the previous 4 months. Although the pancreatic mass was large, there was no evidence of vascular involvement or peritoneal disease and by all criteria was resectable. A somatostatin receptor scintigraphy showed enhanced uptake in the pancreatic tail region and excluded other involved areas. An extended distal pancreatectomy and splenectomy along with resection of the splenic flexure of the colon were performed and the tumor was removed intact. Pathological examination revealed a 14 × 10 × 8 cm solid mass of small cells in the pancreatic tail. Grossly, the mass displayed a glassy cut surface, containing areas of necrosis and hemorrhage. The spleen and four identified lymph nodes were negative for tumor as well as negative for the presence of MCV. Histological evaluation revealed a mitotic, highly active tumor (mitotic count 35 per high power field) with endocrine architecture of solid formations (regular, round nuclei with little cytoplasm). The tumor had immunohistochemically strong staining for synaptophysin, CD56, and in some areas, chromogranin. The tumor cells were strongly positive for cytokeratin 18 with a typically perinuclear granular (dot-like) reaction and also for cytokeratin 20 and the PAN-cytokeratin-specific antibody CK-MNF-116 with a paranuclear “dot-like” pattern (Figure 2A). There was no expression of gastrin, glucagon, insulin, serotonin, somatostatin, TTF-1, or CD117. The proliferative activity (Ki-67) reached approximately 80% (Figure 2B). Evaluating the presence of MCV due to the positivity of the primary tumor, the metastatic tumor was also MCV positive. Based on the clinical, histological, and viral status of the tumor, the diagnosis of a metastatic-MCC in the tail of the pancreas was made. Further histological investigation of the prior removed neuroendocrine breast tumor and the MCC of the left forearm confirmed neuroendocrine origin and identical histology to the previously resected MCC of the left forearm (similar viral profile as well). The patient was referred to oncologists for further evaluation and therapeutic treatment. At 2-year follow-up, the patient was still disease-free, without any complications related to disease progression or recurrence.

## Discussion

MCC is a rare neuroendocrine skin carcinoma with aggressive biological behavior. MCC appears in approximately 50% of cases on the head and neck [1,2]. The second most common sites are the extremities (40%) followed by trunk and genitals (less than 10%) [1,2]. MCC is a carcinoma of the elderly, with a mean age at the time of initial diagnosis of 70 years. It occurs more frequently in immunosuppressed individuals. Many patients who are diagnosed with MCC have a history of other cancers suggesting that MCC may also share etiologic factors with other malignancies [1-3]. Patient symptoms are usually local or secondary to tumor growth or lymph node involvement. Most patients have localized disease at initial presentation (70–80%) [2,4]; the majority develop metastatic disease, either synchronously or metachronously with a frequency varying from 20–75% [4]. It has a penchant for local recurrence and distant metastasis to various sites, including regional lymph nodes, distant skin, lung, liver, testis, and other rare organs such as the pancreas [1-3,5]. Patients with lymph node metastases demonstrate a two- to three-fold higher mortality rate when compared with those without nodal involvement [1].

To our knowledge, there have been only nine previously described cases of MCC affecting the pancreas [2-10]. So far, this is the first case of concomitant pancreatic and breast metastatic MCC disease [2,5] that also evaluates the viral profile of primary and metastatic tumors. There is no information available about the exact incidence of pancreatic metastases of MCC. Secondary neoplasms of the pancreas have been reported to comprise 2–3.9% of pancreatic tumors. In an analysis published by Adsey et al. [6], one case of MCC metastatic to the pancreas was found from a pool of 38 cases of metastatic pancreatic disease. Optimal treatment is based on experience gained from the study of retrospective cases and, especially in the setting of local-regional failure, it remains a major challenge for oncologists. The best current treatment of localized disease appears to be surgery, followed by adjuvant therapy and perhaps systemic chemotherapy [4]. In some cases, definitive radiation therapy could be considered and justified if surgical resection is not possible [4]. The staging of MCC includes a whole-body CT scan because of the frequent high-proliferation index and poor differentiation of the tumor, with the aim of identifying metastatic involvement of soft tissues, sometimes associated with lytic bone lesions [7].

Finally, the recently evaluated role of MCV in the pathogenesis and clinical behavior of MCC should be highlighted. MCV is detected in low levels in normal skin (normal flora), in inflammatory and neoplastic cutaneous diseases, and in non-lesional skin from patients with MCC [11]. MCV is detected in as many as 80–90% of MCCs studied [11] presenting with higher viral load of MCV DNA in MCC compared with benign and other malignant human tissue samples [12]. Its contribution to tumorigenesis is based on the integration of virus before clonal expansion of tumor, the presence of signature viral mutations in tumors, and the expression of viral oncoproteins such as large and small T antigen [11-15]. MCV appears to be associated with certain morphologies of MCC. MCCs with unusual differentiation containing neuroendocrine and non-neuroendocrine elements (complex MCC) or combined MCC with squamous cell carcinoma contained no MCV [13].

MCV-positive and -negative MCCs have differing pathologies. Histologically, MCV-positive MCC tumors have more-regular, rounder nuclei with a smaller cytoplasm than MCV-negative MCC tumors, which have irregular nuclei and a larger cytoplasm [13-15]. The method of choice in identifying MCV is PCR due to its potential to further increase sensitivity for detecting tissue disease by identifying submicroscopic tumor deposits [13].

Table 1 summarizes the previously described cases of MCC metastatic to the pancreas.

**Table 1:** Summary of the described cases of MCC metastatic to the pancreas

**Author**	**Year**	**Presentation**
Safadi R et al. [8]	1996	A 69-year-old woman with chronic lymphocytic leukemia who developed MCC and obstructive jaundice due to pancreatic metastases of the MCC.
Bachmeyer C et al. [7]	2002	A 57-year-old man with MCC on the left lower eyelid. The patient died of generalized carcinomatosis after metastatic MCC invading the stomach and pancreas.
Ouellette JR et al. [2]	2004	A 64-year-old man with obstructive jaundice approximately two years after having a MCC resected from his finger. He underwent a successful pancreaticoduodenectomy with pathology confirming metastatic MCC.
Adsey et al. [6]	2004	One case from a pool of 38 metastatic tumors of the pancreas. No data about the age and the clinical presentation available.
Bachmann J et al. [5]	2005	An 82-year-old woman presented with an abdominal mass, 2 years prior she had an excision done on her eyebrow that was reported as MCC. Final histopathology of the mass was an endocrine carcinoma in the pancreatic tail consistent with metastatic MCC.
Hizawa K et al. [9]	2007	An 85-year-old woman with MCC on the right eyelid. The patient died of an intra-abdominal metastatic MCC that directly invaded the stomach, pancreas and distal duodenum.
Dim DC et al. [3]	2009	A 79-year-old woman with a large pancreatic tail mass and a history of MCC of the upper extremity.
Krejčí K et al. [10]	2010	A 62-year-old man who developed a MCC in the right gluteal region 8 years after combined kidney-pancreas transplantation. The tumor was generalized and metastasized into the pancreatic graft. The patient died 9 months after diagnosis.
Bernstein J et al. [4]	2012	A 56-year-old male presented with a palpable femoral lymph node of a left posterior thigh nodule. Histopathological examination revealed a MCC. A heterogeneous lesion in the pancreatic tail was identified by endoscopic ultrasound.

## Conclusions

MCC is a rare potentially fatal skin tumor with high rates of metastasis and poor survival. We presented a unique case of concomitant MCC in breast and pancreas originating from left forearm. We aim to highlight that MCC has the potential to spread even in unusual organs, such as pancreas or breast, so a diligent follow-up should be applied in patients with MCC.

## Consent

Written informed consent was obtained from the patient for publication of this Case report and any accompanying images. A copy of the written consent is available for review by the Editor-in-Chief of this journal.

## Abbreviations

MCC: Merkel cell carcinoma; MCV: Merkel cell polyomavirus.

## Competing interests

The authors who have taken part in this study declare that they do not have anything to disclose regarding funding or conflict of interest with respect to this manuscript.
